# Investigation of text‐mining methodologies to aid the construction of search strategies in systematic reviews of diagnostic test accuracy—a case study

**DOI:** 10.1002/jrsm.1593

**Published:** 2022-07-31

**Authors:** Hannah O'Keefe, Judith Rankin, Sheila A. Wallace, Fiona Beyer

**Affiliations:** ^1^ Evidence Synthesis Group, National Institute for Health Research (NIHR) Innovation Observatory Newcastle University Newcastle Upon Tyne UK; ^2^ Maternal and Child Health, Population Health Sciences Institute, Faculty of Medical Sciences Newcastle University, Medical School Newcastle Upon Tyne UK; ^3^ Cochrane Incontinence, Evidence Synthesis Group, Population Health Sciences Institute, Faculty of Medical Sciences Newcastle University Newcastle Upon Tyne UK

**Keywords:** information retrieval, literature search, semi‐automation, systematic review, text‐mining

## Abstract

Current methodologies for designing search strategies rely heavily on the knowledge and expertise of information specialists. Yet, the volume and complexity of scientific literature is overwhelming for even the most experienced information specialists, making it difficult to produce robust search strategies for complex systematic reviews. In this case study, we aimed to assess and describe the benefits and limitations of using semi‐automated text‐mining tools for designing search strategies in a systematic review of diagnostic test accuracy. An experienced information specialist designed a search strategy using traditional methods. This strategy was then amended to include additional terms identified by text‐mining tools. We evaluated the usability and expertise required, risk of introducing bias to the search, precision of the search strategy and rated the usefulness of the tools. Thirteen of the 16 investigated tools produced a total of 40 additional terms, beyond those in the original search strategy. This resulted in 11 previously unidentified relevant articles being retrieved. Precision was reduced or remained the same in all cases. After considering all aspects of the investigation we rated each application, with two being ‘extremely useful’, three being ‘useful’, three having ‘no impact’ and eight being ‘not very useful’. Comparative analysis revealed discrepancies between similar tools. Our findings have implications for the way in which these methodologies are used and applied to search strategies. If semi‐automated techniques are to become mainstream in information retrieval for complex systematic reviews, we need tailored tools that fit information specialists' requirements across disciplines.

## 1 BACKGROUND

In healthcare research, many hierarchies place systematic reviews as the highest level of evidence.^1^ Systematic reviews frequently aim to capture all of the available evidence surrounding a given research question.^2^ Information specialists play a fundamental role in the review process from start to completion. The most significant contribution an information specialist will make is the formulation and execution of the search strategy. This strategy will secure the literature base that the review is likely to be founded upon.^2^ It is imperative that this search strategy is able to retrieve as much of the relevant literature as possible so that bias in the review is minimised.^2^, ^3^, ^4^

Information specialists can face many difficulties in designing a complex search strategy. As the volume of health literature expands so does the terminology. This can be highly variable between individuals, institutions and countries, often making it problematic to capture all of the synonyms and acronyms required. Yet without these, studies may be missed and, in turn, compromise the review findings. Even between databases, the thesauri differ; controlled vocabularies in one database will not be the same in another. Information specialists need to have a firm grasp on the review topic to ensure all appropriate subject headings are included in the search strategy.^4^, ^5^ Learning new topics for each review can be time‐consuming, information specialists often spend hours reading around the topic to rapidly accumulate enough knowledge to design the search strategy.^6^ Clinical experts can provide some support for this, yet their individual use of language may differ substantially from others in the field.^5^

Over the last 20 years, the methodologies used by information specialists have largely remained the same despite the increased volume of literature, varied terminology and complexity of review questions.^2^, ^3^ It is increasingly clear that methodologies used by information specialists need to progress. Semi‐automation, the combination of human and machine efforts to perform tasks, has the potential to aid search strategy design by providing a rapid assessment of a subset of the literature.^2^, ^7^, ^8^

Semi‐automation is not routinely used for developing search strategies in systematic reviews despite the volume and complexity of scientific literature used to address some review questions. Of the few information retrieval methodologies that are being introduced, text‐mining tools dominate the field. These tools used alone or in concert, have the potential to substantially reduce the time spent reading to gain an understanding of the subject matter.^8^ For information specialists, it can be hugely valuable to have a rapid assessment which provides a snapshot of the literature. The keywords and phrases that shape the concepts within the subject can be readily identified using text‐mining. This process can help distinguish between informative and uninformative terms which can be a challenging endeavour when unfamiliar with the subject, particularly when terms are rare yet highly influential. These terms can then be incorporated or removed from the search strategy to enhance the performance of the search.^5^

Stakeholders are becoming more interested in what alternative information sources, such as social media, news articles and online forums, can offer. These sources can offer insight into the public's queries, concerns and interests around health care. These are powerful platforms which can be used to aid researchers and policy makers' decisions. Text‐mining can quickly analyse information from these platforms and draw out key concepts which can contribute to the development of the review question and inform the search concepts.^9^ Equally, text‐mining can help to identify misinformation that circulates through these sources via sentence structure analysis, citation chaining and network analysis.^10^ This can assist information specialists in avoiding misleading concepts and terms in the search strategy. By balancing the sensitivity and specificity of a search through text‐mining processes, it can increase the body of evidence for a review. This, in turn, may reduce bias and contribute to the applicability of the findings, resulting in a more robust review. The screening burden will also be reduced as a result of increased specificity of the search which has positive implications for the resources required for the review.

There are contrasting opinions surrounding the use of text‐mining tools as an aid to human input when developing a search strategy. The first argument states that the use of such tools increases the sensitivity of search strategies.^5^ The second argument poses that the inherent biases of using such tools in this context can skew the search strategy.^11^ There is a third argument which sits between these two schools of thought, suggesting that a widespread view cannot be taken across all tools, instead the usefulness of these tools must be considered on an individual basis.^8^

There has been significant work to assess text‐mining tools in the context of search strategy design. However, these in‐depth assessments have widely been conducted by researchers who are familiar with the tools and the conclusions are contradictory.^8^, ^12^, ^13^ These investigations are beneficial to the information specialist community due to the extensive assessment of the tools abilities to contribute positively to the design of the search strategy, yet the naïve user perspective is lost. For many, the usability of these tools is paramount. If they cannot be used in a simple and intuitive way, these tools will not be adopted as a mainstream approach. It is also imperative that information specialists are able to clearly understand the biases that may be introduced to the search strategy by using such tools so that steps can be taken to minimise this.

DTA reviews aim to evaluate whether a diagnostic test (index test) is non‐inferior to known diagnostic tests (reference standard). Designing search strategies for DTA reviews is often challenging as the indexing is often poor or inappropriate, and reporting of methods in the title and abstract can be inconsistent. Although search filters are available, the lack of consistency in subject headings, reporting and terminology can result in relevant literature being missed.^4^, ^14^, ^15^ To this end, in this case study we aimed to assess 16 commonly referenced text‐mining tools and report the intuitiveness of use, potential biases and contributions to the design of a search strategy for a Diagnostic Test Accuracy (DTA) review.

## 2 METHODS

A protocol for this study is available at: https://research.ncl.ac.uk/perinatal-post-mortem-dta/.^4^, ^16^, ^17^ A trained information specialist (HO) designed an initial search strategy in Ovid‐MEDLINE (Data S1). The search used the following concepts: population, index test, reference standard. This was conducted in accordance with standard practice for DTA reviews.^4^, ^18^ The information specialist used a set of five references to explore each text‐mining tool (see below ‘Types of input data’ for further details). These five articles had been previously identified through a scoping search as clinically relevant for inclusion in the DTA review. The review topic is niche, and scoping revealed very little relevant literature. Therefore, these five seed articles were considered to be substantial enough to inform this case study. We used these articles as input to each tool to determine how useful each tool was at identifying terms to include or exclude from the search strategy. Terms identified through the tools were either appended to or removed from the initial search strategy. All amendments were recorded and each iteration of the search was saved as an independent version. All versions of the strategy were subsequently translated to Ovid‐Embase. To ensure consistency in the databases, all versions of the search were run on the same day:

1. MEDLINE (Ovid), 1946 to 16 Dec 2020, searched 18 Dec 2020
2. Embase (Ovid), 1974 to 17 Dec 2020, searched 18 Dec 2020

### 2.1 Types of target studies

The aim of the DTA review was to determine whether non‐invasive or minimally invasive autopsy techniques would be suitable alternatives to traditional autopsy in prenatal and infant death prior to one year of life.^16^, ^19^ The review accepted any study design with a sequential element, assessing the less invasive techniques against the traditional techniques.^4^, ^16^, ^19^

### 2.2 Types of tools

Text‐mining tools commonly cited as being used by information specialists were considered for this investigation. No restrictions were placed on the type of text‐mining, underlying algorithms, accepted input formats or output formats deployed by these tools. Tools from any source or developer were considered. Pay‐for‐services were not fully analysed but were considered for inclusion where sufficient demonstrations or documentation were available to allow the concepts of use to be determined. A total of 16 tools, a mix of web‐based and desktop, were selected:

1. Anne O'Tate^20^
2. BiblioShiny^21^
3. Carrot2^22^
4. CitNetExplorer^23^
5. EndNote^24^
6. Keyword‐Analyzer (formerly TextAlyser)^25^
7. Lingo3G (Carrot)^26^
8. Lingo4G^27^
9. MeSHonDemand^28^
10. PubReMiner^29^
11. TerMine^30^
12. Text Analyzer^31^
13. Tm for R^32^
14. VosViewer^33^
15. Voyant^34^
16. Yale MeSH Analyser^35^

### 2.3 Types of input data

Several approaches were employed based on the tools requirements (Table 1). The following publications were used as a basis for all testing.^36^, ^37^, ^38^, ^39^, ^40^ The publications were downloaded as PDFs and converted into an appropriate format for input into the tools where necessary. Citations were exported and converted to .enw files with EndNote. Web of Science was used to download full reference and citation information as a plain text file. URLs were supplied to the original publications and to the PubMed entries in all cases. PubMed Identification codes (PMID) were sourced for each publication. Finally, for those tools requiring a search string input, the information specialist (HO) designed a short string (separate from the initial search strategies).

As specific database syntax is not applicable to these tools, the string is represented as follows:

**TABLE 1:** Summary of Findings. Groupings, accepted input formats, output styles, risk of bias and usefulness rating for each text‐mining tool

Application	Level of Expertise	Input	Output	Risk of introducing bias	Rating
*Anne O'Tate*	1	Search string for PubMed	Tabular	Moderate	4 Useful
*BiblioShiny*	2	Web of Science, PubMed, Scopus or Dimensions database file	Word cloud Tree map Dynamic graphs	Moderate	2 Not very useful
*Carrot2*	1	Search string for PubMed or Web	Tree map Pie chart	Moderate	3 No Impact
*CitNetExplorer*	2	Web of Science file	Network maps	Moderate	2 Not very useful
*EndNote*	2	Citation files (Multiple formats)	Tabular	Moderate	2 Not very useful
*Keyword‐Analyzer*	1	Paste text	Tabular	Low	3 No Impact
*Lingo3G (Carrot)* ^a^	3	Search string for PubMed or Web	Tree map Pie chart	Moderate^a^	2^a^ Not very useful
*Lingo4G* ^a^	3	Search string for PubMed or Web	Tree map Pie chart	Low^a^	2^a^ Not very useful
*MeSHonDemand*	1	≤10,000‐character paste text	Tabular	Moderate	2 Not very useful
*PubReMiner*	2	Search string for PubMed	Tabular	Moderate	4 Useful
*TerMine*	2	Documents Paste text URL	Tabular In‐text highlighting	Moderate	3 No Impact
*Text Analyzer*	1	Paste text URL	Tabular	Low	5 Extremely useful
*Tm for R*	2	Documents	Tabular Customisable graphics	Moderate	2 Not very useful
*VosViewer*	2	EndNote .enw, RIS or RefWorks citation file. Web of Science, PubMed, Scopus or Dimensions database file. Crossref, PMC, Microsoft Academic, Semantic Scholar, OCC, COCI and Wikidata via search query, DOI or JSON.	Network maps	Moderate	2 Not very useful
*Voyant*	2	Documents Paste text Reference file in XML format URL	Word cloud Frequency trend map Bubble lines Correlation maps Network maps Phrases and keywords in context	Moderate	4 Useful
*Yale MeSH Analyser*	1	≤20 PubMed Identifiers	Tabular	Moderate	5 Extremely useful

*Note*: Level of expertise: 1 = suitable for novice users, 2 = suitable for expert user, 3 = unable to fully evaluate/categorise.

^a^ Based on documentation and demonstrations only.

((‘minimally invasive’ OR ‘minimally‐invasive’ OR ‘non invasive’ OR ‘non‐invasive’)[ti:ab] AND (autops*)[ti:ab] AND (prenatal OR prenate* OR perinatal OR perinate* OR neonatal OR neonate* OR infant)[ti:ab]).

In cases where the use of truncation or quotation marks were not permitted, the following amended representative string was used:

((minimally invasive OR minimally‐invasive OR non invasive OR non‐invasive) [ti:ab] AND (autopsy OR autopsies) [ti:ab] AND (prenatal OR prenate OR prenatal OR prenates OR perinatal OR perinate OR perinates OR neonatal OR neonate OR neonates OR infant) [ti:ab]).

### 2.4 Types of outcome measure

Outcome measures were based on the outputs of the individual tools. Subjective measures of usability were recorded and summarised (Table 2). Terms identified through the tools for inclusion or exclusion from the search strategy were listed (Table 3). The number of retrievals for each version of the search and any loss/gain of relevant publications compared to the original search were documented (Table 4).

**TABLE 2:** Five‐point rating scale used to assess the usefulness of each text‐mining application, with weightings for each criteria to reflect the importance of the aspect for the task

	Scoring criteria (weighting)							
Application	Initial access (1)	Ease of inputting data (2)	Range of input formats (1)	Accessibility of functions (2)	Clarity of outputs (2)	Intuitiveness of use (2)	Impact on the search retrievals (10)	Overall rating (scale: 0–5)
*Anne O'Tate*	5	5	3	3	2	4	4	4
*BiblioShiny*	2	1	2	4	1	3	2	2
*Carrot2*	3	5	3	4	3	5	1	3
*CitNetExplorer*	4	2	2	4	4	2	0	2
*EndNote*	1	4	3	2	5	1	2	2
*Keyword‐Analyzer*	5	3	3	4	3	3	2	3
*Lingo3G(Carrot)* ^a^	1	5	3	4	3	5	0	2
*Lingo4G* ^a^	1	5	3	4	3	5	0	2
*MeSHonDemand*	5	2	1	4	2	3	1	2
*PubReMiner*	5	5	3	3	2	4	5	4
*TerMine*	5	3	2	4	2	3	2	3
*Text Analyser*	5	5	4	5	5	5	5	5
*TM for R*	2	1	5	2	4	1	2	2
*VosViewer*	3	2	2	4	4	2	1	2
*Voyant tools*	5	2	2	1	3	2	5	4
*Yale MeSH Analyzer*	5	5	3	3	4	4	5	5

^a^ Based on documentation and demonstrations, 1 = least useful; 2 = not very useful; 3 = no impact; 4 = useful; 5 = extremely useful.

**TABLE 3:** Suggested terms identified for inclusion in the search strategy, derived from 13 text‐mining applications

Application	Type of text‐mining	Suggested terms
*Anne O'Tate*	Word frequency analysis MeSH identification Phrase frequency analysis	Congenital abnormalities/Diagnostic imaging Fetal diseases/Gestational age/Incisionless Intrauterine Keyhole Whole body imaging
*BiblioShiny*	Co‐location analysis Word frequency analysis Word clustering	Congenital anomaly Conventional autopsy Keyhole autopsy Laparoscopy
*Carrot2*	Topic clustering	Minimally invasive tissue sampling (MITS) PM examination PMCT
*EndNote*	Keyword frequency analysis	Congenital abnormalities Fetal diseases/Gestational age/Laparoscopy Whole body imaging
*Keyword‐analyzer*	Word frequency analysis Topic clustering	Babies/Baby Fetal care High‐resolution imaging Intrauterine death Keyhole autopsy MR imaging MRI‐derived diagnostic Pregnancy complications Radiologist Spontaneous vaginal delivery
*MeSHonDemand*	Mesh identification	Gestational age/Radiologist/Radiology
*PubReMiner*	Word frequency analysis	Congenital abnormality/Fetal diseases/Infant Intrauterine Laparoscopic Newborn Pregnancy complications/ Infant, premature, diseases/ Radiography Radiology Robotic Whole body imaging
*Termine*	C‐value tagging	Antemortem information Conventional autopsy Cross‐sectional imaging External examination Gestational age Laparoscopic MR imaging PM examination Radiologist Robotic system
*Text Analyzer*	Word frequency analysis	Antepartum Babies/Baby Conventional autopsy Feticide Keyhole autopsy Laparoscopic Less invasive MR imaging Newborn PM examinations Umbilical Umbilicus Virtuopsy
*Tm for R*	Word frequency analysis	Intrapartum Intrauterine Laparoscopic Radiologist Radiology Virtobot
*VosViewer*	Word clustering	Conventional autopsy PM examination PM MRI
*Voyant*	Co‐location analysis Word frequency analysis	Conventional autopsy Echopsy Keyhole autopsy Laparoscopy Newborns PM dissection Radiologist Robotic Virtobot Virtuopsy
*Yale MeSH Analyser*	Mesh identification	Congenital abnormalities/Fetal diseases/Gestational age/Infant/Laparoscopy/Whole body imaging/

*Note*: Subject heading (MeSH).

Abbreviations: CT, computed tomography; MR(I), magnetic resonance (imaging); PM, post mortem.

**TABLE 4:** Number of retrieved studies from search strategies built using the suggested terms from each text‐mining application, demonstrating the total number retrieved, the difference from the original search and the number of relevant unique retrievals in that subset of additional records

	Ovid‐MEDLINE			Ovid‐Embase		
Application	Retrieved	Difference^a^	Relevant (*n* = 10)	Retrieved	Difference^a^	Relevant (*n* = 5)
*Anne O'Tate*	590	161	4	574	113	2
*BiblioShiny*	497	68	1	573	112	2
*Carrot2*	429	0	0	461	0	0
*EndNote*	499	70	2	471	10	1
*Keyword‐Analyzer*	453	24	1	595	134	3
*MeSHonDemand*	453	16	0	476	15	0
*PubReMiner*	632	203	9	785	324	5
*Termine*	446	17	1	476	15	0
*Text Analyzer*	578	149	8	582	121	3
*Tm for R*	444	15	1	475	14	0
*VosViewer*	429	0	0	461	0	0
*Voyant*	676	247	9	597	136	3
*Yale MeSH Analyser*	625	196	9	663	202	5

^a^ Difference between the number of studies retrieved from the amended search and the original search.

### 2.5 Data collection and analysis

#### 2.5.1 Assessment of risk of introducing bias

Authors considered sources of bias due to the features of the tools and how methods used in this investigation could introduce bias. Each tool was rated as at low, moderate or high risk of bias. Sources of bias that were taken into consideration included selection bias (through the choice of seed papers and/or search results), confirmation/observer bias (possibility of subconsciously finding patterns or conclusions from outputs), publication bias (skew of publications with positive results), measurement bias (output measures, e.g., score or counts), sample bias/data representativeness (due to broad or narrow seed set and/or search results), institutional bias (institutions publishing more frequently on a given topic than others), and exclusion bias (excluding features of the data, e.g., information from tables). The features of the tools were related to these sources of bias, for example, those that accept a search string as input may introduce sample bias if the resulting search is too broad or too narrow and cannot be controlled within the application.

#### 2.5.2 Data synthesis

Ease of use was assessed for each application. This included observations of the ease of inputting data to the tools and if inputs could be appended after the initial input, the accessibility of functions within the tools, any complications during the process, the clarity of the outputs, whether they could be modified and whether they could be saved. Tools were grouped by ease of use, groupings were as follows: 1 = suitable for novice users, 2 = suitable for expert users, 3 = unable to fully evaluate/categorise (due to paywall limiting access to full functionality) (Table 1).

The number of retrievals for each search strategy version was recorded and compared to the initial search. Where a difference was found, the records lost/gained were inspected to identify which records were appropriate for inclusion in the DTA review. The precision of the original search and each of the amended searches was calculated as:

$$\text{Included studies}/\left(\text{included studies}+\text{excluded studies}\right)$$

The collated data were used to score each application. Ratings were given as a numerical value of 1–5 with these corresponding explanations:

1. Least useful—difficult to use with no impact on the literature retrieval (0 unique articles).
2. Not very useful—difficult to use and very little impact on the literature retrieval (1–3 unique articles).
3. No impact—moderate performance across both areas, or high in one and low in the other (4–6 unique articles).
4. Useful—easy to use and moderate impact on the literature retrieval (7–10 unique articles).
5. Extremely useful—very easy to use with extensive impact on the literature retrieval (11+ unique articles).

The score was derived based on the assessment of usability and the impact each tool had on the development of the search strategy. Those tools that were not available to test sufficiently had their ratings based on the information available in documentation and demonstrations. The judgement criteria were weighted to reflect the importance of each criterion, with impact on the search weighted the strongest.

Comparative analysis of the outputs was conducted in the following combinations:

1. CitNetExplorer + BiblioShiny
2. Voyant + Keyword‐Analyzer (TextAlyser) + Text Analyzer

These tools accepted sufficiently similar input formats and conducted the same type of text‐mining analysis. The outputs were compared by percentage similarity. Tools with reduced percentage similarity were investigated further to identify reasons for this discrepancy.

## 3 RESULTS

Of the 16 tools chosen for review, 14 were available to test sufficiently and two were analysed based on documentation alone.^20^ This is due to the tools requiring subscriptions as pay‐for services. However, where documentation or demonstrations were available, the concepts of the pay‐for services were noted, specifically Lingo3G and Lingo4G which are pay‐for desktop downloads.^26^, ^27^ EndNote was an exception and was tested fully as it is readily available through many institutional subscription services.^24^ Table 1 provides a summary of the findings and demonstrates the inputs, outputs, bias assessment and relative scale of usefulness for each application. We found that it took no longer than 20 min to run each tool, however, the time to decipher the outputs varied from 10 min to 1 h.

### 3.1 Original search results

The original search strategy (Data S1) retrieved a total of 429 records from Ovid‐MEDLINE and 461 from Ovid‐Embase. After the removal of duplicate records, there were 653 unique records that underwent title and abstract screening. Of these, 135 matched the eligibility criteria at this stage and were taken forward for full‐text screening. Full texts were screened against the eligibility criteria for the DTA review [available at: https://research.ncl.ac.uk/perinatal-post-mortem-dta/]. A total of 76 records were identified for inclusion in the review, 62/76 were identified in Ovid‐MEDLINE and 45/76 were identified in Ovid‐Embase.

### 3.2 Included search terms

**FIGURE 1:**
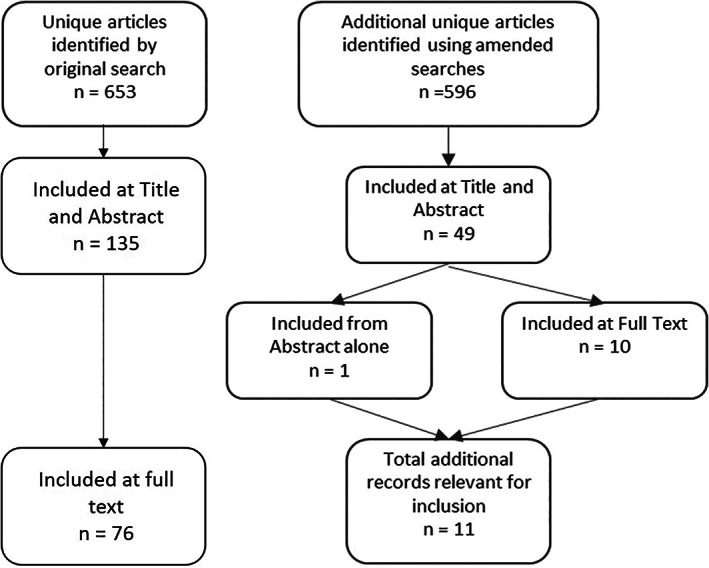
Flow chart of the screening process for additional articles identified through the amended searches

As we found that CitNetExplorer is designed purely for conducting citation analysis, we did not investigate this tool for potential search terms. A total of 40 suggestions were found from the outcomes of the remaining 13 tools (Table 3). These suggestions were worked up into appropriate MeSH and Emtree subject headings, as well as free text terms. The original search strategy was amended with each of the sets of terms, providing 13 unique strategies for comparison against the original. This was repeated in both Ovid‐MEDLNE and Ovid‐Embase and all search strategies were run on the same day. In total, 596 unique articles were identified using the amended searches. This was in addition to the 653 unique records identified by the original search (Figure 1). The titles and abstracts of the additional records were screened initially, with 49 articles deemed relevant at this stage. In five cases it was not possible to retrieve the full texts and interlibrary loans were considered beyond the scope of this investigation. In any case, it was suspected that it would not be possible to isolate the population from four of these, however, one was clearly eligible for inclusion from the abstract alone and was taken forward. The full texts of the remaining 44 articles were retrieved and screened against the inclusion and exclusion criteria for the review. Of these, 10 met the eligibility criteria for inclusion, six of which were derived from the same study cohort. (See Data S2 for a list of excluded studies).

This gave a total of 11 additional articles (10 full texts and one abstract from six studies) which have been deemed relevant for inclusion in the DTA review (Figure 1). Six of these were identified through the Ovid‐MEDLINE search only, whereas one of the articles was found through Ovid‐Embase only, the remaining four were found in both databases. The individual searches retrieved varying numbers of these 11 articles in each database (Table 4). The amendments made in accordance with suggestions from PubReMiner and Yale MeSH Analyzer retrieved the highest number, with both retrieving nine from Ovid‐MEDLINE and five from Ovid‐Embase.^29^, ^35^ Amendments in accordance with suggestions from Voyant and Text Analyzer also yielded higher numbers in Ovid‐Medline, nine and eight, respectively, and Ovid‐Embase, with three each.^31^, ^34^ Conversely, the amended search strategies based on suggestions from Carrot2, MeSHonDemand and VosViewer did not retrieve any of the 11 additional articles from either database.^22^, ^28^, ^33^ Amended search strategies based on suggestions from Anne O'Tate, BiblioShiny, EndNote, Keyword‐Analyzer, TerMine and TM for R all retrieved relatively few of the additional articles (Table 4).^20^, ^21^, ^24^, ^25^, ^30^, ^32^

No terms were identified by the tools for exclusion from the search. However, terms for exclusion are not offered by these tools. Instead, this is a subjective assessment by the information specialist.

### 3.3 Precision of the searches

The original searches demonstrated 15% and 10% precision for Ovid‐MEDLINE and Ovid‐Embase, respectively. As anticipated, the precision was reduced for all of the amended Ovid‐MEDLINE searches. This was also the case for the majority of Ovid‐Embase searches, with the exception of three which showed no change in precision (Table 5).

**TABLE 5:** Precision of the amended search strategies, demonstrating the total number of studies eligible for inclusion in the review, the total number excluded from the review and the precision of this search, calculated as included studies/(included + excluded studies), and whether precision was increased or decreased compared with the precision of the original search

	Ovid‐Medline				Ovid‐Embase			
Application	Includes	Excludes	Precision	+/−	Includes	Excludes	Precision	+/−
*Anne O'Tate*	66	524	0.11	‐	47	527	0.08	‐
*BiblioShiny*	63	434	0.13	‐	47	526	0.08	‐
*Carrot2*	62	367	0.14	‐	45	416	0.10	NC
*EndNote*	64	435	0.13	‐	46	425	0.10	NC
*Keyword‐Analyzer*	63	390	0.14	‐	48	547	0.08	‐
*MeSHonDemand*	62	391	0.14	‐	45	431	0.09	‐
*PubReMiner*	71	561	0.11	‐	50	735	0.06	‐
*Termine*	63	383	0.14	‐	45	431	0.09	‐
*Text Analyzer*	70	508	0.12	‐	48	534	0.08	‐
*Tm for R*	63	381	0.14	‐	45	430	0.09	‐
*VosViewer*	62	367	0.14	‐	45	416	0.10	NC
*Voyant*	71	605	0.11	‐	48	549	0.08	‐
*Yale MeSH Analyser*	71	554	0.11	‐	50	613	0.08	‐

*Note*: NC ‐ no change, + higher precision, − lower precision.

### 3.4 Risk of introducing bias

The main concern around bias in the tools is the limitation of the input formats. In most cases these are restricted to specific sources, character counts or coding formats, as seen with MeSHonDemand which is limited to a maximum of 10,000 characters. This narrows the possibility of assessing relevant texts, either they may not be available through a specific platform such as PubMed or Web of Science, or character restriction may mean that only a small portion of the text can be analysed. Where inputs are required to be in ASCII or UTF‐8 encoded format, input of texts in another encoding format may result in words being misinterpreted or ignored by the tools' algorithms, biasing the outputs. Similarly, there is a possibility of bias if the user is not able to correctly adhere to complex input requirements. The predominant form of bias resulting from these issues is selection bias, where the selection of input texts is not representative of the wider literature.^11^, ^41^ Exclusion bias is also a risk factor when using these tools as important features of the data may be missed by the underlying algorithms. Due to this, the majority of these tools can be considered as having a moderate risk of introducing bias. Those where text can be directly pasted into the tool without character or word limits have a lower risk as there are no complications with formatting. The risk of introducing bias for each tool has been listed in Table 1.

There is the possibility of bias being introduced by the chosen seed papers. Four of the five studies are centred around imaging techniques, this may skew the results of the analysis as other techniques, such as endoscopic biopsy, would be misrepresented in this sample.^36^, ^37^, ^38^, ^40^ The populations covered by these studies are representative of the perinatal population being addressed in the DTA review. Therefore, the population terms identified through this investigation may be more reliable than the intervention terms. There is also a possibility of institutional bias in these studies. Four of the five studies are UK based,^36^, ^37^, ^38^, ^39^ the remaining study is based in Switzerland.^40^ These countries are high‐income and do not represent low and middle‐income countries. Studies with negative results are less likely to reach publication stage, increasing the risk of publication bias.^41^ However, we have tried to combat this by using seed papers which report either positive or negative results. The concerns around introducing bias from the publications may also be more broadly applied to results retrieved through search functionalities within the tools. It is difficult to control many of these factors, particularly when conducting searches through the tools as the underlying algorithms used to find related publications and generate results are often a black box, and further exploration is needed to fully understand the impact of this and whether the algorithms generate bias.

### 3.5 Usability and usefulness score

Anne O'Tate is a web‐based tool which accepts a search string input.^20^ Brackets can be used to define more complex searches; however, the use of truncation or quotations is not permitted. Therefore, the string void of truncation and quotations was used for this investigation. PubMed is the source that this tool searches and some limits can be applied before searching. The initial output from Anne O'Tate is a list of relevant articles returned from PubMed. Other outputs can be retrieved by clicking on the links to the left of the page. These include word lists, phrase lists with importance scores, MeSH terms and clusters by topics. It is possible to adapt the search string after it has been run by using the ‘details’ tab. Whilst the tool is simple to use, the outputs are not always intuitive, and they cannot be saved.^20^ As Anne O'Tate is a freely available web tool with a simple search string input, it scored highly for these criteria.^20^ It scored moderately for the range of inputs and accessibility of functions within the application. However, it did not score as well for the clarity (understandability) of the outputs. Overall, Anne O'Tate was given a rating of ‘4: Useful’ as the overall usability scored reasonably and the amendments suggested by the tool had a reasonable impact on the retrieval of literature that was relevant to the review question (Table 2).

BiblioShiny is a freely available RStudio‐Shiny package.^21^ This tool will be displayed as a web interface to the user. However, the user needs a basic knowledge of starting tools from within RStudio to be able to open the web interface. The input formats for this tool are Web of Science, Scopus or Dimensions files, and PubMed API searching.^21^ For this investigation, a Web of Science file containing the full reference and citations was used. An attempt was made to use the inbuilt PubMed API connection, however, this failed to retrieve the records. The BiblioShiny web interface has an impressive selection of outputs. Disappointingly, the large majority of these do not work. In addition, error messages occur which are designed to alert the program designer to an issue, these are not comprehensible to users with little or no knowledge of computer programming and can appear daunting. It also appears that only tabular data can be saved from the web interface. This is frustrating as the graphical outputs can hold valuable information. Screenshots may be taken to capture the information, although the user may need to take several zoomed in screenshots to capture the granularity of the graphical outputs. No guidance or explanation is given for any of the outputs which means at times they are hard to interpret. It feels as though this tool has been designed for a more expert audience which may make it too challenging for inexperienced users. BiblioShiny^21^ generally scored low across the usability criteria, although a reasonable rating was given for the accessibility of functions within the application. The amendments in response to the suggestions had little impact on the retrieval of relevant literature. Overall a rating of ‘2: Not very useful’ was given for this tool (Table 2). However, experienced users may gain more from the tools functions and benefit from the outputs.

CitNetExplorer is built by the same designers as VosViewer and is also available as a desktop tool which requires Java installation.^23^ However, where VosViewer is sometimes blocked by the Windows operating system, CitNetExplorer does not face the same issue. CitNetExplorer accepts Web of Science files which means the user must source the required publications in Web of Science and download a Tab delimited file before being able to use this application.^23^ For this investigation, a Web of Science file containing the full reference and citations was used. The outputs are a network map and table which aim to link citation information and, like VosViewer, the output can be saved. Whilst this output may not be directly applicable to generating a search strategy, it could be useful when thinking about reference searching. CitNetExplorer^23^ did not contribute to the amendments for the search strategy, therefore it scored the lowest possible rating in this category. It did score moderately across the usability criteria; however, this was not enough to award a rating of more than ‘2: Not very useful’ (Table 2).

EndNote is a pay‐for application;^24^ however, many institutions will have a subscription to the service. EndNote is a citation manager which incorporates references from many different platforms. In order to perform frequency analysis on the imported citations, the user must follow the path below:

Tools → Subject bibliography → Key words → OK → Select All → OK → Layout → Terms → Subject Terms Only → remove ^p^p from Suffix → Term Count → Descending → OK.

This will count the occurrence of terms in the Key Word field and provide frequency data. To apply this to other fields simply change the third step. For this investigation, an EndNote file was generated, containing references for each of the relevant papers.

The pitfalls of this approach are that it is only applicable after an EndNote library has been collated and that not all entries will have keywords associated with them. However, it may be useful for checking that no significant terms have been missed from the search strategy. The output is a list of terms and their frequency which can be printed or saved in a rich text .rtf, HTML .htm or plain text .txt file format.^24^ A low score was given for initial access to the tool as it is a pay‐for‐service and the intuitiveness of use.^24^ The remaining usability criteria scored reasonably, with clarity of outputs being awarded the highest score. However, the impact on the literature retrieval was low, resulting in an overall rating of ‘2: Not very useful’ (Table 2).

Keyword‐Analyzer is a web‐based tool which was recently updated in 2020, previously named TextAlyser.^25^ The tool accepts pasted text as its input and does not appear to have a limit on the number of characters or length of the pasted text. For this investigation, the text of all five papers, extracted from .PDF files, was pasted into the application. The outputs given by Keyword‐Analyzer are tabular with each new table sitting in a new tab area. This makes it easy to navigate across the tabs and look at each new table, except where a table is particularly long and spans several pages. Tables revolve around various measures of word and phrase frequency, topic identification and sentence structure which can be beneficial when designing a search strategy. Unfortunately, the majority of the outputs cannot be saved which means it is not practical to compare multiple runs of analysis. The only output which is exportable is the Keyword density evaluation, covering word and phrase frequency, which can be exported as a csv file.^25^ However, this does not list the words, simply a numbering system in place of actual text. As a free access web‐based application, Keyword‐Analyzer^25^ achieved the highest available score for initial access. However, it scored moderately across the remaining criteria, including impact on the literature retrieval. Therefore, a rating of ‘3: No impact’ was awarded (Table 2).

Little distinguishes the Lingo3G(Carrot), Lingo4G and Carrot2 tools in terms of availability, inputs and outputs.^22^, ^26^, ^27^ All three are available as desktop tools. However, Lingo3G and Lingo4G are pay‐for services and have not been examined fully in this investigation, instead documentation and demonstrations were used as the basis for this narrative.^26^, ^27^ As all three platforms are created by the same company, they appear to be very similar in nature. Java installation is needed to ensure the desktop tools work correctly. For this investigation, the online platform for Carrot2 was used to avoid Java issues. A search string is accepted as the input format and a choice is offered for web searching or PubMed searching. Here, the search string including truncation and quotations was used in Carrot2.^22^ The outputs are given as folders, tree maps or pie charts. Each of these denotes a selection of themes and the number of publications or web results occurring under each theme. Each publication or web result may belong to one or more themes. The outputs also have links to each of the websites for the publications or web results which makes it easy to view the content and download it if desired. Both tree map and pie chart outputs can be saved and automatically downloaded in .jpg file format.^22^ These tools are graphically appealing; however, the limited input types and outputs mean these tools are only suitable for giving an overarching view of the subject area. Perhaps they are best suited to the initial stages of exploratory analysis.

Carrot2^22^ scored relatively well across the criteria for usability, with ease of input and intuitiveness of use scoring the highest. Unfortunately, the suggestions given by this tool did not result in any of the relevant literature being retrieved, reducing the overall rating of this tool to ‘3: No impact’ (Table 2). Neither Lingo3G(Carrot) or Lingo4G were available to test sufficiently. Therefore the decisions made in the scoring process were based on the available documentation and demonstrations.^26^, ^27^ For this reason, initial access scored very low. Conversely, ease of inputting data and intuitiveness of use scored highly. The remaining usability criteria resulted in a modest score. Neither tool contributed to the search strategy due to inability to test the tools, perhaps unfairly achieving the lowest score in this category. The similarities between these tools meant that both achieved the same score for each criterion and both were awarded a rating of ‘2: Not very useful’ (Table 2).

MeSHonDemand is a web‐based tool with a simple ‘paste text’ input. Unfortunately, this input is limited to ≤10,000 characters.^28^ It is generally aimed at analysis of titles and abstracts or a short manuscript. This investigation utilised the abstracts from each relevant paper as input. This might raise concerns that such a short input may miss substantial information. MeSHonDemand compares the input with PubMed articles to find similar content.^28^ From this it calculates a set of plausible MeSH terms, highlights related content in the abstract text entered and supplies a list of the top 10 related articles in PubMed along with links to the articles. The user may have to repeat the analysis with several abstracts in order to gain a comprehensive list of MeSH terms for inclusion in their search strategy. The output can be saved in a plain text .txt file format which can make it easy to compare outputs from multiple analyses. The highest available score was given for initial access as this is a freely available web application.^28^ However, range of input formats and impact on the retrieval of relevant literature both scored very low. The remaining criteria had moderate scores. This resulted in MeSHonDemand receiving a rating of ‘2: Not very useful’ (Table 2).

PubReMiner is a web‐based tool which takes a search string as input. If the user requires a more complex search, brackets can be used to group similar terms together and some limits can be applied.^29^ Here, the search string including truncation and quotations was used. This is a relatively simple input system which works effectively to search PubMed. The outputs are tabular with number of publications and frequency data for each term. The tables also have selection boxes or hyperlinks that allow the user to add particular terms to their search string and narrow the return. A subset of the outputs have links to PubMed which will list all publications related to that term.^29^ Whilst this is quite a simple tool to use, the outputs are not visually appealing and can seem cluttered. Due to space constraints, some of the tables are positioned underneath others which also makes it difficult to navigate the page. The advantage of this tool is the list of words and MeSH terms that are generated. It is not immediately obvious which column refers to the number of journals and which refers to the total frequency of a term, making it difficult to quickly assess the relevance of words and MeSH terms. However, this can be deciphered as the journal count will be equal to or lower than the frequency count. One benefit is that outputs can be saved in a plain text .txt file format. PubReMiner^29^ achieved moderate to high scores for every criterion except clarity of outputs. This was let down simply by the visual appeal and organisation of the outputs which made them difficult to comprehend. An overall rating of ‘4: Useful’ was awarded (Table 2).

TerMine is a web‐based tool with several input formats. Users are able to paste text into a box, upload up to 2 MB of plain text .txt files and .PDF files or input URLs for up to 2 MB of HTML or PDF content. If the user requires larger inputs a batch test can be submitted, although this is processed in a queue and may take a few days to return, depending on how busy the service currently is.^30^ The limitations of the inputs are that any text, pasted or uploaded as files, must be ACSII encoded. Most file types are either ASCII or UTF‐8 encoded but the user must check before inputting. Problems occur when using URLs for PDFs that have originated from the PMC library, no results are returned or occasionally copyright limitation forbids this action.^30^ One way of bypassing this is to paste the text into TerMine, although this may not be practical with a larger volume of text and any copyright limitations must be adhered to. For this investigation, an attempt was made to enter URLs, however, this failed for the majority of papers due to these limitations. Therefore, .PDF files were uploaded, although this could only be achieved one at a time. The initial output given is a corpus of text with highlighted phrases. This can be adjusted by the user to set a threshold based on the C‐Score which will alter the highlighting of the text with phrases above the threshold in red and those below in blue.^30^ There is the option for the user to see the results in a tabular or text form which will open in a separate window. This provides the highlighted phrases in rank order with a C‐Score. The text output provides these as one continuous string which makes it difficult to separate the phrases, the table is more readable. C‐Scores are based on the algorithm used to classify and rank phrases and have multiple decimal places. They are not easy to discern and if the user is unable to comprehend the C‐Scores then any threshold limit put in place is arbitrary. Rank may be a more useful measure for understanding the output. Unfortunately, it is not possible to save any of the outputs for this application. TerMine^30^ scored moderately across four criteria. Initial access was given the highest available score. The remaining three criteria were awarded lower scores, including the impact on the retrieval of relevant literature. This resulted in a rating of ‘3: No impact’ (Table 2).

Text Analyser is a simple web‐based application. It has two input options, pasting text and URLs.^31^ For this investigation, the text of all five papers, extracted from .PDF files, was pasted into the application. Text Analyser's output consists of phrase occurrences in a tabular format. Each table presents phrases with a particular number of words. The table with the highest number of words in a phrase is at the top of the page and the remaining tables follow in descending order.^31^ There is also a table of unfiltered word counts at the end of the page. Whilst this is a simple tool with basic tabular outputs, some of the phrases detected in each table are repetitive and differ by a single word at one end of the phrase. Users are unable to save outputs from this application. Text Analyser^31^ was awarded the highest available score for every criteria, except range of input formats which scored one stage down. This provided an overall rating of ‘5: Extremely useful’ (Table 2).

Like BiblioShiny, TM for R is a freely available RStudio package. TM for R runs within the RStudio desktop interface.^32^ Whilst it is relatively simple to use, it is not recommended for individuals without R programming experience. Plain text files can be used as input which is not particularly beneficial as files would need to be converted to this format before use. Instead, another package that is specifically built to read .PDF file formats was used for this investigation.^42^ Those with R programming experience will be able to use other packages to convert file formats or enable direct loading of other file formats, as achieved here, but this is not achievable for novice users. The TM package can determine word frequencies and associations. These outputs will be displayed on the console in tabular formats.^32^ The use of secondary packages such as word cloud and ggplots can be used to transform the TM outputs to graphical summaries.^43^, ^44^ Generally, the use of the TM package may be best for developing tools which could be used by a wider audience. TM for R scored well in range of input formats and clarity of outputs; however, this is dependent on the user's ability to manipulate the code.^32^ Otherwise, low scores were awarded due to the need for prior programming knowledge. It is notable that we have experience with R and RStudio, yet still found this package to score poorly as it had little impact on the retrieval of relevant literature. An overall rating of ‘2: Not very useful’ was awarded (Table 2).

VosViewer is a free desktop tool which requires Java to be installed. A web‐based version is available if the user has Java support, however, this is not easy to launch and may be blocked by some systems.^33^ It is notable that certain web browsers, such as Google Chrome, no longer support Java applets and the web version will not run on these browsers. Furthermore, the web version will only accept JSN or VosViewer map files, limiting its applicability in this context. Similarly, Windows will, in some circumstances, attempt to block the desktop version. In general, VosViewer is not a particularly intuitive application. The user may search a small selection of databases by author or title to find inputs. Other input options include uploading references in EndNote .enw or .ris file format or documents in Web of Science, PubMed, Scopus or Dimensions.^33^ Whilst these input formats are extensive, it is also the main pitfall of the application. In order to prepare an EndNote file in .enw or .ris format, the user must export the file using the EndNote Export or RefMan (RIS) Export output style, respectively. This may prove difficult to those unfamiliar with EndNote file formats, although in the context of information retrieval these are commonly used file formats and should not pose a difficulty. For this investigation, a .ris file of the relevant citations was used. The tool allows database searching; however, it is not possible to perform complex searches which limits the returns. Furthermore, if the user wishes to analyse a subset of relevant publications, they must source these from one of the mentioned databases which can be inconvenient. All inputs must be uploaded at once otherwise the data will be overwritten with any additions. When utilising the database search function data will only be extracted from the title and/or abstract not the full texts.^33^ This may be acceptable as many users will be building their search strategies based on title, abstract and keyword searching. The most promising aspect of this tool is the network output. The networking algorithm clusters words or short phrases into categories and colours the outputs to distinguish them. This makes it possible for the user to identify themes which can help to define the concepts of the search strategy. It is possible to adjust the outputs with a series of radio buttons and sliders. Three views of the output are available which allows the user to select their preference and it is possible to save all views. VosViewer^33^ achieved low to moderate scoring for each of the usability criteria. A very low score was given for impact on the retrieval of literature as no relevant articles were obtained as a result of this tools suggested terms. Overall, a rating of ‘2: Not very useful’ was given (Table 2).

Voyant is a web‐based tool which allows the user to upload multiple publications, paste text, input reference files as XML or supply URLs. All inputs must be uploaded at one time, there is no capacity to add or remove individual files after the initial upload. For this investigation, pasted text from the .PDF files was used as input.^34^ One of the key benefits of this tool is the range of graphical outputs which can be somewhat customised by the user through sliders and input boxes. Interestingly, each panel of the tools interface can be customised to show different outputs. This is beneficial as some of the outputs are not particularly intuitive and it allows users to pick the outputs that are most easily interpretable to them. Whilst this is a great feature of the tool, it is not obvious how to use the customisation function. This means many users may not know about its existence and therefore only interact with the default outputs, missing much of the functionality available through this application. Similarly, it is possible to alter the default options for each output, save the outputs as image files, clear the input by returning to the homepage, specify the interface language and export references, URLs, HTML and Spyral notebooks.^34^ The widgets that enable these functions have small icons that appear when hovered over, meaning none of these functions are obvious to the user. The most pressing disadvantage of this tool is the handling of .PDF formats, notably those from Elsevier. Publications from Elsevier contain information about the citations which places words such as ‘http’ and ‘elsevier.com’ at the most frequent. This skews the analysis and makes it difficult to interpret true results. However, specific words or phrases can be added to the stopword dictionary and removed from the analysis, again through a somewhat hidden functionality within the application. It was noted that in Voyant .PDF file uploads are analysed individually, whereas pasted text is analysed as a single corpus.^34^ The outputs are affected by this, for example, word frequency analysis produces multiple counts of the same word when analysing individual files, whereas it produces a single count for a word when analysing a combined corpus. It was decided that pasted text input was warranted for this investigation to enable comparative analysis to be conducted. However, this may have consequences when identifying terms for inclusion in the search strategy. There are notable differences with a pasted input, Bubblelines and trend analysis are representative of the corpus as a whole and are not able to distinguish patterns in the individual documents.^34^ Additionally, correlation analysis is not retrievable. Correlation analysis identifies how term frequencies are synchronised throughout the document. This may be beneficial in identifying co‐located terms. Voyant^34^ scored very highly for initial access and impact on the retrieval of relevant literature. Interestingly, it achieved relatively low scores for the remaining criteria, with the lowest for accessibility of functions within the application. Yet, Voyant still achieved an overall rating of ‘4: Useful’ due to its higher score for impact (Table 2).

Yale MeSH Analyzer is a web‐based tool which takes ≤20 PubMed Identifiers as its input.^35^ Although not explicitly stated, the tool will also accept PMC Identifiers. Here, the PubMed identifiers of the relevant papers were used. It can be time‐consuming to acquire the identifiers and some of the user's relevant articles may not be available through the PubMed platform. The output is in tabular format, each article is summarised and any MeSH terms and author keywords used for that article in PubMed are listed.^35^ Users have the option to customise the output slightly and to either present it as a HTML table which opens on the web page or to download the table automatically as an Excel file. Unfortunately, the option to save as an Excel file has to be specified before the analysis is run and no option to save in any format is provided after. Moderate to high scores were achieved in all categories, with the highest available score for initial access, ease of inputting data and impact on the retrieval of relevant literature. This resulted in an overall score of ‘5: Extremely useful’ for Yale MeSH Analyzer^35^ (Table 2).

### 3.6 Comparative analysis

CitNetExplorer's citations network was compared to BiblioShiny's histography network.^21^, ^23^ Both tools provided very similar outputs. The only identifiable difference was that the citation for Thayyil et al.^38^ was missing from the BiblioShiny output. BiblioShiny does not register citations which do not have any network connections, whereas CitNetExplorer can be programmed to show or hide unlinked citations.^23^ However, with only five citations as input, the outputs were too small to draw any meaningful inferences from this comparison.

The outputs for Voyant, Keyword‐Analyzer and Text Analyser could not be directly compared.^25^, ^31^, ^34^ Text Analyser does not remove stop words from the corpus, whereas Voyant and Keyword‐Analyzer do. Therefore, stop words were removed manually from the Text Analyser outputs so that comparison could be conducted. The comparative analysis was limited to the 50 highest frequency words for each application. It would be expected that all three tools would give the same outputs. That is, the outputs would include the same words in the top 50, and the frequency of each word within the text would be counted the same regardless of the tool used.

Only 20% (*n* = 10) of terms had the same frequency listed in the outputs from all three tools. There were 58% (*n* = 29) of terms where the frequency was different in the outputs of each application (range: 1 to 22 counts). Interestingly, Keyword‐Analyzer did not include 22% (*n* = 11) of the terms that were present in the top 50 from the other two tools, having alternative terms instead. Unfortunately, it was not possible to find details regarding the underlying algorithms used in Keyword‐Analyzer.^25^ Therefore, further exploration of the discrepancies between the tools was not possible. However, it is thought that this may be due to the way that the tools handle reference lists within publications.

It was decided that a separate comparison of outputs from Voyant and Text Analyser was warranted.^31^, ^34^ In this case, both tools included the same terms in their top 50 terms list. The tools listed the same frequency for 74% (*n* = 37) of the terms, but listed 26% (*n* = 13) of terms as having different frequencies within the texts (range: 1 to 6 counts).

## 4 DISCUSSION

A total of 40 terms were suggested for inclusion in the search strategy, generated by 13 of the tools (range: 3–13 terms). None of the tools suggested terms which could have been removed from the search strategy (e.g. those occurring at very high frequency but with minimal specificity). This resulted in 11 additional articles which are relevant to the DTA review being retrieved from the databases compared to the results of the original search. Perhaps unsurprisingly, those tools producing the highest number of suggestions returned the most relevant articles, whereas those with the lowest number of suggestions did not return any of the relevant articles. Three of the tools were deemed at low risk of introducing bias with the remainder at moderate risk. However, two of the low risk tools were not available to assess sufficiently (Table 1).^26^, ^27^ Two of the tools were rated as being Extremely Useful.^31^, ^35^ A further three tools were rated as Useful,^20^, ^29^, ^34^ with three rated as having No Impact.^22^, ^25^, ^30^ The remaining eight tools were rated as Not very useful,^21^, ^23^, ^24^, ^26^, ^27^, ^28^, ^32^, ^33^ again two of these were not available to assess sufficiently (Table 1).^26^, ^27^ It should be noted that this case study was designed with novice users in mind, therefore, those with greater experience of these tools or high levels of computer literacy may find the outputs more useful than we have scored them. We have indicated which tools are most suitable for novice or expert users in Table 1.

### 4.1 Extremely useful tools

Text Analyzer^31^ produced the highest number of suggested terms, with a total of 13. This resulted in 8 and 3 relevant unique retrievals from Ovid‐MEDLINE and Ovid‐Embase, with precision of 12% and 8%, respectively (Table 5). This tool was thought to be at low risk of introducing bias and was given an overall rating of Extremely Useful (Table 1). Text Analyzer showed a good percentage similarity with one tool (Voyant) from the comparative group, but not with the other (Keyword‐Analyzer).

Yale MeSH Analyser^35^ provided six suggestions, resulting in nine and five relevant retrievals from Ovid‐MEDLINE and Ovid‐Embase, with precision of 11% and 8%, respectively (Table 5). This is the highest number of retrievals, matched by only PubReMiner (Table 4). Yale MeSH Analyser was considered as having a moderate risk of introducing bias but was awarded a rating of Extremely Useful (Table 1).

### 4.2 Useful tools

Anne O′Tate^20^ provided eight suggestions for inclusion in the search strategy. These resulted in four relevant additional returns from Ovid‐MEDLINE and two from Ovid‐Embase, with precision of 11% and 8%, respectively (Table 5). It was deemed at moderate risk of introducing bias and awarded an overall rating of Useful (Table 1).

Voyant^34^ provided 10 suggested terms for inclusion in the search strategy. This resulted in nine relevant retrievals from Ovid‐MEDLINE and three from Ovid‐Embase, with precision of 11% and 8%, respectively (Table 5). It was thought that Voyant had a moderate risk of introducing bias and it was given an overall rating of Useful (Table 1). When compared to similar tools, Voyant showed a good percentage similarity with Text Analyzer, but not Keyword‐Analyzer.

PubReMiner^29^ provided 12 terms as suggestions for inclusion in the search strategy. This resulted in 9 and 5 relevant articles being returned from Ovid‐MEDLINE and Ovid‐Embase, with precision of 11% and 6%, respectively (Table 5). It was given a rating of Useful and thought to be at moderate risk of introducing bias (Table 1).

### 4.3 Key issues with current tools

There are issues with accessibility for those tools that require Java support.^23^, ^33^ Each tool may require a different version of Java, newer versions will overwrite the old and some tools will fail because of this. It may also affect other tools on the user's computer or the user may have limitations to what they can download, especially with institutionally owned machines.

The learning curve can be steep for some of the tools. Novice users may find this too demanding and it negates the idea that these tools will save the user time and resources. Many of the tools have PubMed searches as input features.^20^, ^21^, ^22^, ^26^, ^27^, ^29^ However, due to the limitations of the tool programming interface (API) it is not possible to send extensive requests to PubMed.^45^ Similarly, the tools are designed with a minimal interface so users are unable to build up complex searches as they would via the PubMed interface. Some of the tools require overly specific input formats which make it difficult for the user to ensure their data is correctly formatted. None of the tools investigated here allow the user to append additional inputs to their analysis. Once an input has been given the user must overwrite this if they wish to add or remove content. This is aggravated when it is not possible to save outputs from the analysis.^20^, ^21^, ^25^, ^30^, ^31^, ^35^ The user must take screenshots to ensure they can review the outputs at a later time or use them to compare between analyses. Furthermore, the outputs presented from many of the tools are visually unappealing and, in some cases, difficult to interpret.

As demonstrated by the comparative analysis performed in this investigation, it is clear that not all tools perform as expected. This means that information specialists need to be cautious about the tools they use and have a firm understanding of exactly what is happening in the background of each one. This is difficult as many of the tools do not have sufficient instruction or explanation to understand this. These issues, either singularly or in combination, may impede the use of the tools and prevent them being adopted as common practice. It is recommended that information specialists take the time to familiarise themselves with the tools. There are YouTube tutorials and publications readily available which may be easier to process than the tools accompanying manuals.

Certain inherent biases are unavoidable given the nature of use. For example, information specialists are often given a small sample of papers from clinical experts which will not reflect the entire body of literature. Results based on this sample will be biased as the tools will only analyse the content of what is given to them.^11^ A lack of knowledge on the part of the information specialist can also subconsciously introduce bias, whether this is lack of knowledge regarding the subject or regarding the tool of choice. However, this inherent bias does not negate the importance of this investigation. If anything, this investigation aims to reduce bias by providing a level of understanding to help information specialists choose an appropriate tool for their task.

The methods of this investigation have been conducted as robustly as possible. However, there is still the possibility that bias has been introduced. This is particularly true for selection bias, where the five included studies used to assess the tools may not have truly represented the breadth and depth of the existing literature.^11^ However, the studies utilised for this investigation include one protocol without results,^40^ three studies with positive results^36^, ^37^, ^38^ and one study with negative results,^39^ minimising any risk of publication bias. The unconscious decisions made during this investigation may have introduced observer bias.^46^ This could sway the analysis in favour of our unconscious assumptions. In addition to this, two of the tools were assessed using only the documentation and guidance that was available. Due to the self‐promotional nature of this documentation, it may have skewed the scoring in a positive direction. The results for these two tools should be interpreted with caution as this approach may have introduced anchoring bias.^47^ Authors have endeavoured to reduce bias by predefining the methods within the protocol.^16^

### 4.4 Applicability to the investigative question

The information specialist (HO) who designed the original search strategy used in this case study was already knowledgeable about the topic of the DTA review.^4^, ^16^, ^19^ The original search was already extensive, covering many of the terms that would have otherwise been identified using these tools. Perhaps the use of these tools may be of more benefit when the information specialist is less familiar with the topic.^8^ One point of note is the lack of terms identified for exclusion from the search strategy. This may also have been influenced by the topic knowledge of the information specialist. Equally, the use of additional papers for exploring the tools would have resulted in different outcomes, possibly leading to the identification of terms for exclusion. It is important to re‐iterate that this investigation is subjective and exploratory. Whilst the authors have taken care to pre‐specify and adhere to the investigative methods laid out in a detailed protocol, the results are open to interpretation.^16^ Therefore, no certainty can be given that these findings will apply to all information specialists when developing a search strategy. The aim of this investigation was to provide an assessment of the benefits and detriments of using text‐mining tools to aid search strategy development in DTA reviews. To this end, the tools and methods used here are sufficient to investigate this.

This investigation has drawn similar conclusions to previous studies in this area. Glanville, et al. 2018., explored a range of text‐mining tools to determine if there are any tools which may be beneficial for information specialists, particularly in the context of Health Technology Assessments.^8^ Whilst this investigation has been conducted from a novice user's perspective and has covered a different range of tools for a different purpose, the same conclusions have been drawn. There is agreement that there is a lack of tailored tools for information specialists and those that are available should be used cautiously and with careful consideration.^8^ There is also agreement that those that require programming skills may be less accessible to information specialists. Here, it was noted that TM for R and BiblioShiny fall under this umbrella due to the need for R programming experience.^21^, ^32^

Thomas, et al. 2011., considered the use of computational tools throughout the systematic review process.^11^ Regarding the use of text‐mining tools to aid search strategy development, they noted *Its limitation is a function of its strength: it expands the review in favour of the literature that uses the same language as the documents that have already been found. This method will not identify cross‐disciplinary research very well. For example, one discipline, or body of research might refer to ‘health services’, whereas other people might describe them as ‘health care provision’. This method on its own will not assist the reviewer in identifying literatures that use different words to describe the same concepts*.^11^ This resonates highly with the inherent sources of bias that have been discussed in this investigation. Due to the limitation of the inputs and the functionalities within the tools, there is no method of retrieving alternative description other than MeSH subject headings unless using EndNote to either divide records into batches based on particular controlled vocabularies or export the vocabularies to analyse.

Hausner, et al. 2016., compared objective and subjective approaches to search strategy design. They describe the objective approach as the use of tools and the subjective approach as the use of standard methods.^5^ This is very similar to the analysis conducted here, where tools were used to enhance a search strategy designed using standard methods. Hausner, et al. 2016., concluded that the objective approach resulted in increased sensitivity and similar precision of the search.^5^ This investigation did not evaluate the sensitivity of the search and therefore can neither agree nor disagree with this finding. However, it can be concluded that the enhanced search strategies returned more than the original search and, in many cases, detected relevant literature that would otherwise have been missed, suggesting an increase in sensitivity. In contrast to the findings of Hausner, et al. 2016., for the majority of cases in this investigation, the precision was reduced compared to that of the original search. This is because the amended search strategies return additional irrelevant literature in substantially higher volumes than additional relevant literature. Whilst there is the benefit of finding extra literature, it comes at a cost for screening time.

Paynter, et al. 2021., compared the use of text‐mining tools vs. usual practice for designing the search strategies in a series of reviews. They found that in most cases the searches designed using text‐mining tools were less sensitive than traditional searches but the process time was faster.^13^ Again, this investigation did not evaluate sensitivity but the findings suggest it would be increased rather than decreased. Paynter, et al. 2021., also noted that the use of text‐mining tools for complex topics retrieved additional records that weren't identified by traditional searches.^13^ This is similar to our findings, where additional records were identified, but raises questions about their sensitivity analysis.

## 5 CONCLUSIONS

### 5.1 Implications for systematic reviews and evaluations of healthcare

This case study has demonstrated that the use of text‐mining tools can aid search strategy development and enhance the number of relevant retrievals in DTA reviews. Despite the original search strategy being designed by an information specialist with good knowledge of the topic, the tools were able to suggest terms which had otherwise been overlooked. The inclusion of these terms had varying degrees of success when attempting to find additional literature that was relevant to the DTA review.^16^, ^19^ Additional relevant records were found in many cases, however, the benefit of retrieving these needs to be weighed against the resources required to screen the volume of additional literature, most of which was not relevant to the DTA review (Figure 1). The impact that these additional relevant records have on the findings of the DTA review were not considered here. The decision to use supporting tools should be made in the context of time‐on‐task, and screening burden relative to the number of extra studies identified for inclusion. It should not be based upon whether the extra studies included in the review have impacted the outcome of the review findings as this introduces bias.

Whilst some tools performed better than others, the authors believe that individuals should consider multiple aspects before choosing which, if any, tool would suit their needs.^8^ Firstly, information specialists should consider how familiar they are with the topic of the review.^4^ If they are very familiar with it, it may be sufficient to have the search strategy peer‐reviewed with or without needing to use text‐mining tools. Peer‐review may be more time efficient than using multiple tools, particularly if the search strategy is already extensive. If the information specialist believes that using text‐mining would be advantageous, they should then consider the formats of their existing literature set and how these match with the input formats of the tools. Similarly, the information specialist's preferences for tabular or graphical outputs should be considered alongside the type of analysis they require. As established previously, there is a risk of introducing bias when using these tools, therefore, the degree of acceptable risk should also be considered. Finally, the information specialist needs to consider how much time is available to invest in this exercise and whether they have funding to purchase tools or licences. The comparative analysis here has highlighted that even though tools appear to be performing the same analysis this is not always the case.^25^, ^31^, ^34^ It is important that caution is taken in this respect. These text‐mining tools can help but should not be used as the sole source to inform the development of a search strategy.^2^

A search strategy for a standard systematic review of interventions may benefit less from the use of text mining tools than review types with more complex search requirements, such as DTA or Prognostic reviews. However, text mining tools may be useful in situations where terminology varies between regions or countries, or where clinical experts are not readily available to advise on suitable terms and the information specialist is less familiar with the nuances of the subject.

### 5.2 Implications for methodological research

One of the key limitations of these tools is that they are mostly not tailored to the needs of information specialists.^8^ This is demonstrated by the specificities of the input formats, many of which are not coherent with formats regularly used by information specialists, and the intricacies of the functions within the tools. If information specialists are to use text‐mining tools as part of their mainstream practices then several aspects need to be considered.^5^, ^8^, ^11^ Firstly, these tools must have reasonable licence fees or be freely available to download or use via a web interface. Given the difficulties with Java support, particularly in an institutional setting, it is important that the need for Java support is limited or non‐existent. Secondly, information specialists are not usually computational experts and have very little time available, therefore, it is vital that the interface of these tools is well defined with a clear layout and easy to use with little or no experience. This investigation has demonstrated that this is not always the case, with some tools requiring a steep learning curve. This need for ease of use includes the functions available within the tools, which should reflect the skills and expertise of information specialists. A clear example of this is the inclusion of PubMed searches; information specialists are highly skilled at developing bibliographic database searches and are used to incorporating Boolean logic, truncation and wildcards, this should be reflected in the application. The outputs are perhaps the most important aspect of any text‐mining application, this applies to any user, not just information specialists. Outputs should be clear, aesthetically appealing and downloadable for documentation and further analysis purposes. Many of the tools examined here did not have the option to download the outputs. Finally, it was very evident that there is a need for help and explanation to be provided within the tools. This may take the form of help pages, pop‐ups or in‐line text. Whilst most of the tools gave some text to aid the user via manuals or publications, there was a lack of immediate explanation within the tool around the functions and the analysis that was being run. It was not clear if the tools removed stop words, how punctuation was handled or if the reference lists of articles were included in the analysis. This has an impact on the outputs and could potentially skew a search strategy if not handled cautiously. This investigation highlights the need for development or adaptation of text‐mining tools specifically for information specialists.^8^

## AUTHOR CONTRIBUTIONS

All authors contributed to the study conception and design. Material preparation, data collection and analysis were performed by Hannah O'Keefe. The first draft of the manuscript was written by Hannah O'Keefe and all authors commented on subsequent versions of the manuscript. All authors read and approved the final manuscript.

## FUNDING INFORMATION

The authors did not receive support from any organisation for the submitted work. Judith Rankin is part‐funded by the National Institute of Health Research Applied Research Collaboration North East and North Cumbria.

## CONFLICT OF INTEREST

Authors have no relevant financial or non‐financial interests to disclose.

## Supporting information


**Appendix S1 Supp 1.** The original search strategies for MEDLINE and Embase, constructed in Ovid.

**Appendix S2 Supp 2.** Studies excluded from the review, with reasons for exclusion.

## Data Availability

The data supporting the findings of this study are publicly available in PubMed at https://pubmed.ncbi.nlm.nih.gov/.
