# Supplementary material for: Investigation of text‐mining methodologies to aid the construction of search strategies in systematic reviews of diagnostic test accuracy—a case study
Source: Res Synth Methods. 2022 Jul 31;14(1):79–98. doi: 10.1002/jrsm.1593 (PMC10088010; doi:10.1002/jrsm.1593)
Supplement: Supplementary file 1 — Appendix S1 Supp 1. The original search strategies for MEDLINE and Embase, constructed in Ovid. [file JRSM-14-79-s002.docx]

**Supplemental material 1.**

The original search strategies for Medline and Embase, constructed in Ovid.

**Medline:**

1. exp Pregnancy Outcome/

2. "stillbirth*".ti,ab,kw,kf.

3. abortion*.ti,ab,kw,kf.

4. Gestational Age/

5. exp Infant Death/

6. exp Infant Mortality/

7. Fetal Mortality/

8. exp Fetal Death/

9. neonat*.ti,ab,kw,kf.

10. infant.ti,ab,kw,kf.

11. (f?etal or f?etus).ti,ab,kw,kf.

12. prenat*.ti,ab,kw,kf.

13. perinat*.ti,ab,kw,kf.

14. Perinatal Death/

15. Perinatal Mortality/

16. exp Abortion, Spontaneous/

17. exp Abortion, Induced/

18. (termination adj3 pregnancy).ti,ab,kw,kf.

19. Sudden Infant Death/

20. or/1-19

21. Autopsy/

22. necropsy.ti,ab,kw,kf.

23. autops*.ti,ab,kw,kf.

24. post?mortem*.ti,ab,kw,kf.

25. Forensic Pathology/

26. "cause of death"/ or fatal outcome/

27. or/21-26

28. 20 and 27

29. non?invasive.ti,ab,kw,kf.

30. "minimally invasive".ti,ab,kw,kf.

31. Magnetic Resonance Imaging/

32. "percutaneous biopsy".ti,ab,kw,kf.

33. "endoscop* biop*".ti,ab,kw,kf.

34. ultrasound.ti,ab,kw,kf.

35. "f?etal free DNA".ti,ab,kw,kf.

36. Amniocentesis/

37. virtops*.ti,ab,kw,kf.

38. "virtual autops*".ti,ab,kw,kf.

39. "verbal autops*".ti,ab,kw,kf.

40. exp Umbilical Cord/

41. exp Placenta/

42. exp Placenta Diseases/

43. exp Tomography, X-Ray Computed/

44. ultrasonography/ or exp ultrasonography, prenatal/

45. exp Biopsy/

46. "chorionic villus".ti,ab,kw,kf.

47. amniocentesis.ti,ab,kw,kf.

48. (MRI or CT).ti,ab,kw,kf.

49. exp Prenatal Diagnosis/

50. or/29-49

51. 28 and 50

52. exp "Sensitivity and Specificity"/

53. sensitivity.tw.

54. specificity.tw.

55. ((pre-test or pretest) adj probability).tw.

56. post-test probability.tw.

57. predictive value$.tw.

58. likelihood ratio$.tw.

59. cross sectional.tw.

60. Cross-Sectional Studies/

61. Diagnostic accuracy.ti,ab,kw,kf.

62. or/52-61

63. 51 and 62

**Embase:**

1. exp pregnancy outcome/

2. "stillbirth*".ti,ab,kw.

3. abortion*.ti,ab,kw.

4. gestational age/

5. exp child death/

6. exp infant mortality/

7. fetus mortality/

8. exp fetus death/

9. neonat*.ti,ab,kw.

10. infant.ti,ab,kw.

11. (f?etal or f?etus).ti,ab,kw.

12. prenat*.ti,ab,kw.

13. perinat*.ti,ab,kw.

14. perinatal death/

15. exp perinatal mortality/

16. exp induced abortion/

17. exp abortion/

18. exp pregnancy termination/

19. (termination adj3 pregnancy).ti,ab,kw.

20. sudden infant death syndrome/

21. or/1-20

22. autopsy/

23. necropsy.ti,ab,kw.

24. autops*.ti,ab,kw.

25. post?mortem*.ti,ab,kw.

26. forensic pathology/

27. "cause of death"/

28. fatality/

29. or/22-28

30. 21 and 29

31. non?invasive.ti,ab,kw.

32. "minimally invasive".ti,ab,kw.

33. exp nuclear magnetic resonance imaging/

34. "percutaneous biopsy".ti,ab,kw.

35. "endoscop* biop*".ti,ab,kw.

36. ultrasound.ti,ab,kw.

37. "f?etal free DNA".ti,ab,kw.

38. exp amniocentesis/

39. virtops*.ti,ab,kw.

40. "virtual autops*".ti,ab,kw.

41. "verbal autops*".ti,ab,kw.

42. exp umbilical cord/

43. exp placenta/

44. exp placenta disorder/

45. exp computer assisted tomography/

46. exp fetal ultrasound monitor/ or exp ultrasound guided fine needle aspiration/ or ultrasound/

47. exp fetus echography/

48. exp biopsy/

49. "chorionic villus".ti,ab,kw.

50. amniocentesis.ti,ab,kw.

51. (MRI or CT).ti,ab,kw.

52. exp prenatal diagnosis/

53. or/31-52

54. 30 and 53

55. exp "sensitivity and specificity"/

56. sensitivity.tw.

57. specificity.tw.

58. ((pre-test or pretest) adj probability).tw.

59. post-test probability.tw.

60. predictive value$.tw.

61. likelihood ratio$.tw.

62. *Diagnostic Accuracy/

63. cross sectional.tw.

64. cross-sectional study/

65. or/55-64

66. 54 and 65
