# Supplementary material for: Investigation of text‐mining methodologies to aid the construction of search strategies in systematic reviews of diagnostic test accuracy—a case study
Source: Res Synth Methods. 2022 Jul 31;14(1):79–98. doi: 10.1002/jrsm.1593 (PMC10088010; doi:10.1002/jrsm.1593)
Supplement: Supplementary file 2 — Appendix S2 Supp 2. Studies excluded from the review, with reasons for exclusion. [file JRSM-14-79-s001.docx]

**Supplemental material 2.**

Full texts were assessed for eligibility against prespecified criteria. A total of 34 studies were excluded at this stage. The reasons for exclusion are listed below:

Review articles (n = 3)

Systematic review articles (n = 2)

Not the population of interest (n = 8)

Could not isolate the population of interest (n = 12)

Wrong reference standard (n = 3)

Index test delivered in the ante-mortem period (n = 5)

Reference standard conducted before the index test (n = 1)

| Review articles (n = 3) | 1. Arthurs OJ, van Rijn RR, Taylor AM, Sebire NJ. Paediatric and perinatal postmortem imaging: the need for a subspecialty approach. Pediatric radiology. 2015;45(4):483-90.  2. Dedouit F, Otal P, Costagliola R, Loubes Lacroix F, Telmon N, Rouge D, et al. [Role of modern cross-sectional imaging in thanatology: a pictorial essay]. Application a la thanatologie de l'imagerie en coupe: revue iconographique. 2006;87(6):619-38.  3. Mateen FJ, Kalter HD. Review: Verbal autopsy for neurological diseases. American Journal of Tropical Medicine and Hygiene. 2012;86(2):237-9. |
| --- | --- |
| Systematic review articles (n = 2) | 1. Hostiuc S, Rusu MC, Negoi I, Hostiuc M. Sensitivity of autopsy versus computer tomography and coronary angiography in detecting the prevalence and morphological characteristics of myocardial bridging. A meta-analysis. Rechtsmedizin. 2017;27(4):315.  2. Thayyil S, Chandrasekaran M, Chitty LS, Wade A, Skordis-Worrall J, Bennett-Britton I, et al. Diagnostic accuracy of post-mortem magnetic resonance imaging in fetuses, children and adults: a systematic review. European journal of radiology. 2010;75(1):e142-8. |
| Not the population of interest (n = 8) | 1. Antunez S, Grevent D, Boddaert N, Vergnaud E, Vecchione A, Ferrant-Azoulay O, et al. "Perimortem" total body CT-scan examination in severely injured children: an informative insight into the causes of death. International journal of legal medicine. 2020;134(2):625-35.  2. Desai N, Aleksandrowicz L, Miasnikof P, Lu Y, Leitao J, Byass P, et al. Performance of four computer-coded verbal autopsy methods for cause of death assignment compared with physician coding on 24,000 deaths in low- and middle-income countries. BMC Medicine. 2014;12(1):20.  3. Jackowski C, Dirnhofer S, Thali M, Aghayev E, Dirnhofer R, Sonnenschein M. Postmortem diagnostics using MSCT and MRI of a lethal streptococcus group A infection at infancy: a case report. Forensic science international. 2005;151(2):157-63.  4. Palmiere C, Binaghi S, Doenz F, Bize P, Chevallier C, Mangin P, et al. Detection of hemorrhage source: the diagnostic value of post-mortem CT-angiography. Forensic science international. 2012;222(1):33-9.  5. Ross S, Ebner L, Flach P, Brodhage R, Bolliger SA, Christe A, et al. Postmortem Whole-Body MRI in traumatic causes of death. American Journal of Roentgenology. 2012;199(6):1186-92.  6. Shirota G, Gonoi W, Ikemura M, Ishida M, Shintani Y, Abe H, et al. The pseudo-SAH sign: an imaging pitfall in postmortem computed tomography. International journal of legal medicine. 2017;131(6):1647-53.  7. Uto K, Nagao M, Kikuchi N, Onizuka H, Yoshizawa S, Hagiwara N, et al. An Autopsy Case of Arrhythmogenic Right Ventricular Cardiomyopathy - Radiological/Pathological Comparison. Circulation journal : official journal of the Japanese Circulation Society. 2020;84(6):1045.  8. Van Rooden S, Maat-Schieman MLC, Nabuurs RJA, Van Der Weerd L, Van Duijn S, Van Duinen SG, et al. Cerebral amyloidosis: Postmortem detection with human 7.0-T MR imaging system. Radiology. 2009;253(3):788-96. |
| Could not isolate the population of interest (n = 12) | 1. Ganapathy SS, Yi Yi K, Omar MA, Anuar MFM, Jeevananthan C, Rao C. Validation of verbal autopsy: determination of cause of deaths in Malaysia 2013. BMC public health. 2017;17(1):653.  2. Anon J, Remonda L, Spreng A, Scheurer E, Schroth G, Boesch C, et al. Traumatic extra-axial hemorrhage: correlation of postmortem MSCT, MRI, and forensic-pathological findings. Journal of magnetic resonance imaging : JMRI. 2008;28(4):823-36.  3. Hong TS, Reyes JA, Moineddin R, Chiasson DA, Berdon WE, Babyn PS. Value of postmortem thoracic CT over radiography in imaging of pediatric rib fractures. Pediatric Radiology. 2011;41(6):736-48.  4. Joubert J, Bradshaw D, Kabudula C, Rao C, Kahn K, Mee P, et al. Record-linkage comparison of verbal autopsy and routine civil registration death certification in rural north-east South Africa: 2006-09. International journal of epidemiology. 2014;43(6):1945-58.  5. Mobley CC, Boerma JT, Titus S, Lohrke B, Shangula K, Black RE. Validation study of a verbal autopsy method for causes of childhood mortality in Namibia. Journal of Tropical Pediatrics. 1996;42(6):365-9.  6. Mpimbaza A, Filler S, Katureebe A, Kinara SO, Nzabandora E, Quick L, et al. Validity of verbal autopsy procedures for determining malaria deaths in different epidemiological settings in Uganda. PloS one. 2011;6(10):e26892.  7. Rodriguez L, Reyes H, Tome P, Ridaura C, Flores S, Guiscafre H. Validation of the verbal autopsy method to ascertain acute respiratory infection as cause of death. Indian journal of pediatrics. 1998;65(4):579-84.  8. Schifman AG, Scribano P. The prevalence and necessity of obtaining postmortem skeletal surveys in children under the age of 2 who die in the hospital setting. Annals of Emergency Medicine. 2011;58(4):S200-S1.  9. Shelmerdine SC, Langan D, Hutchinson JC, Hickson M, Pawley K, Suich J, et al. Chest radiographs versus CT for the detection of rib fractures in children (DRIFT): a diagnostic accuracy observational study. The Lancet Child and Adolescent Health. 2018;2(11):802-11.  10. Sieswerda-Hoogendoorn T, Soerdjbalie-Maikoe V, de Bakker H, van Rijn RR. Postmortem CT compared to autopsy in children; concordance in a forensic setting. International journal of legal medicine. 2014;128(6):957-65.  11. Westphal SE, Apitzsch J, Penzkofer T, Mahnken AH, Knuchel R. Virtual CT autopsy in clinical pathology: feasibility in clinical autopsies. Virchows Archiv : an international journal of pathology. 2012;461(2):211-9.  12. Yen K, Lovblad KO, Scheurer E, Ozdoba C, Thali MJ, Aghayev E, et al. Post-mortem forensic neuroimaging: Correlation of MSCT and MRI findings with autopsy results. Forensic Science International. 2007;173(1):21-35. |
| Wrong reference standard (n = 3) | 1. Han H, Tao W, Zhang M. The dural entrance of cerebral bridging veins into the superior sagittal sinus: an anatomical comparison between cadavers and digital subtraction angiography. Neuroradiology. 2007;49(2):169-75.  2. Menahem S, Venables AW. Anomalous left coronary artery from the pulmonary artery: A 15 year sample. British Heart Journal. 1987;58(4):378-84.  3. Yu S, Haughton VM, Sether LA, Wagner M. Comparison of MR and diskography in detecting radial tears of the anulus: A postmortem study. American Journal of Neuroradiology. 1989;10(5):1077-81. |
| Index test delivered in the ante-mortem period (n = 5) | 1. Atik E, Cury P, Albuquerque AMT. Aortic atresia with aortopulmonary window and interrupted aortic arch, simulating common arterial trunk: A case report. International Journal of Cardiology. 1998;66(2):217-21.  2. Lange LW, Sahn DJ, Allen HD. Cross-sectional echocardiography in hypoplastic left ventricle: Echocardiographic-angiographic-anatomic correlations. Pediatric Cardiology. 1980;1(4):287-99.  3. Lo RN, Lau KC, Aung-Khin M. Aortic atresia with complete transposition. British heart journal. 1987;57(5):483-6.  4. Redington AN, Rigby ML, Ho SY, Gunthard J, Anderson RH. Aortic atresia with aortopulmonary window and interruption of the aortic arch. Pediatric cardiology. 1991;12(1):49-51.  5. Trounce JQ, Fagan D, Levene MI. Intraventricular haemorrhage and periventricular leucomalacia: Ultrasound and autopsy correlation. Archives of Disease in Childhood. 1986;61(12):1203-7. |
| Reference standard conducted before the index test (n = 1) | 1. Julsrud PR, Weigel TJ, Edwards WD. Angiographic determination of ventricular morphology: correlation with pathology in 36 hearts with single functional ventricles. Pediatric cardiology. 1997;18(3):208-12. |
